# The New Concept of Univentricular Heart

**DOI:** 10.3389/fped.2014.00062

**Published:** 2014-07-07

**Authors:** Carla Frescura, Gaetano Thiene

**Affiliations:** ^1^Cardiovascular Pathology, Department of Cardiac Thoracic and Vascular Sciences, University of Padua Medical School, Padua, Italy

**Keywords:** aortic atresia, double inlet ventricle, mitral atresia, pulmonary atresia, single ventricle, tricuspid atresia, univentricular heart

## Abstract

The concept of univentricular heart moved from hearts with only one ventricle connected with atria [double inlet ventricle or absent atrioventricular (AV) connection] to hearts not amenable to biventricular repair, namely hearts with two ventricles unable to sustain separately pulmonary and systemic circulations in sequence. In the latter definition, even hearts with one hypoplastic ventricle are considered “functional” univentricular hearts. They include pulmonary/aortic atresia or severe stenosis with hypoplastic ventricle, and rare conditions like huge intramural cardiac tumors and Ebstein anomaly with extreme atrialization of right ventricular cavity. In this setting, the surgical repair is univentricular with “Fontan” operation, bypassing the ventricular mass. In other words, functionally univentricular heart is a condition in which, after surgery, only one ventricle sustain systemic circulation. Univentricular hearts (double inlet or absent AV connection) almost invariably show two ventricular chambers, one main and one accessory, which lacks an inlet portion. The latter is located posteriorly when morphologically left and anteriorly when morphologically right. As far as double inlet left ventricle, this is usually associated with discordant ventriculo-arterial (VA) connection (transposition of the great arteries) and all the blood flow to the aorta, which takes origin from the hypoplastic anterior right ventricle, is ventricular septal defect (bulbo-ventricular foramen) dependent. If restrictive, an aortic arch obstruction may be present. Double inlet left ventricle may be rarely associated with VA concordance (Holmes heart). As far as double inlet right ventricle with posterior hypoplastic left ventricular cavity, ventriculo-arterial connection is usually of double outlet type; thus the term double inlet–outlet right ventricle may be coined. Absent right or left AV connection may develop in the setting of both d- or l-loop, whatever the situs. In this condition, the contra-lateral patent AV valve may be either mitral or tricuspid in terms of morphology and the underlying ventricle (main chamber) either morphologically left or right. Establishing the loop, whatever right or left (also called right or left ventricular topology), is a fundamental step in the segmental-sequential analysis of congenital heart disease.

## Introduction

The segmental, sequential approach used for the analysis and categorization of the heart’s malformations has indubitably improved the diagnosis and classification of complex congenital heart disease (CHD).

De la Cruz (1) and Van Praagh (2–4) first emphasized the advantage to consider the malformed hearts in terms of atrial, ventricular, and arterial components, however to our mind, the merit of systematic categorization and clinical application has to be assigned to the English School of Anderson and co-workers (5–11).

**Figure 1:**
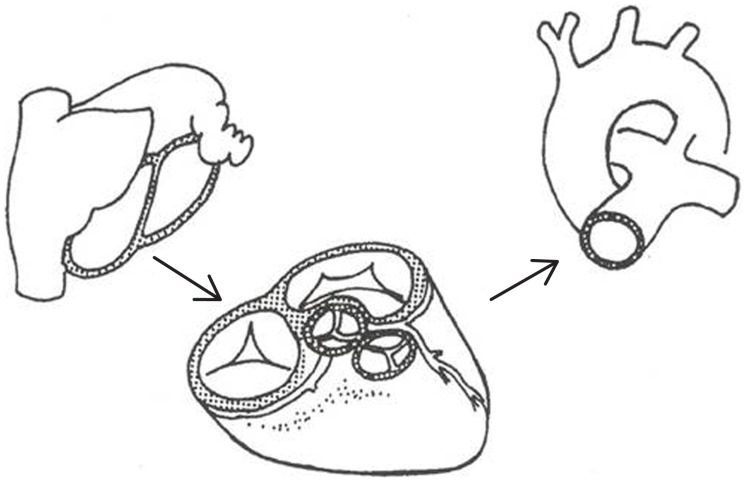
**Segmental analysis of congenital heart disease**. The normal heart consists of three segments: atria, ventricles, and the great arteries connected each other at atrioventricular and ventriculo-arterial junctions [partially modified from Refs. (12, 13)].

While approaching the diagnosis of any CHD, you should consider the heart as a three floor building. The atria are the platform, the ventricles the first floor, and the great arteries the second floor, connected each other through valve orifices and divided by septa (Figure 1) (12, 13).

The three segments are separated by two junctions, named atrioventricular (AV) and ventriculo-arterial (VA). The first connects each atrium with the corresponding ventricle and the second connects each ventricle with the appropriate artery. Normally, each junction possesses two distinct valves: mitral and tricuspid valves at AV and pulmonary and aortic valves in the VA junctions.

The AV connection is *biventricular* when each atrium connects separately with one ventricle. The biventricular AV connection is concordant when the right atrium connects with the right ventricle and the left atrium connects with the left ventricle, discordant when the right atrium connects with the left ventricle and the left atrium connects with the right ventricle, whatever be the situs, either solitus or inversus. In the setting of isomerism of right and left appendages, the AV connection cannot be defined as concordant or discordant but biventricular and the topology of the ventricles (d or l-loop) should be added.

In the presence of biventricular AV connection, the right and left ventricles are of normal morphology and good size, each being constituted by inlet, outlet, and apico-trabecular components. The presence of two separated valves or of a common AV valve does not change the basic connection, whether concordant or discordant.

**Figure 2:**
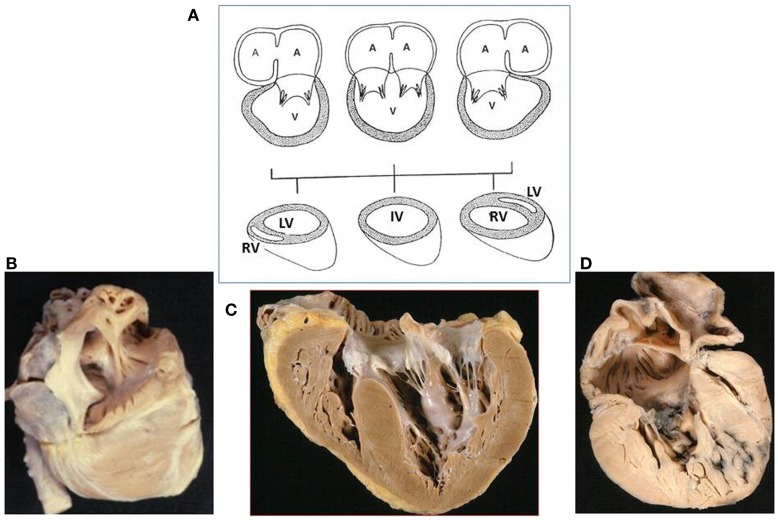
**Univentricular atrioventricular connection**. **(A)** The atrioventricular connection is *univentricular* when both atria drain mostly into a single ventricular chamber through two patent or a common atrioventricular valves (double inlet connection) or through a single valve in case of absent right or left connection. Note the anterior position of the right ventricle and the posterior position of the left ventricle when the main chamber is of left or right morphology, respectively. In the absence of ventricular septum, the main ventricular chamber is of indeterminate morphology (IV). **(B)**. Absent right atrioventricular connection (tricuspid atresia), viewed from the right atrium: no orifice is present at the right atrial floor. **(C)** Double inlet ventricle: two separate valves drain into a main ventricular chamber of left morphology: note the hypoplastic right ventricle. The right atrioventricular valve is overriding the interventricular septum. **(D)** Absent left atrioventricular connection (mitral atresia): no orifice is present at the left atrial floor and the left ventricle is slit-like. A, atrium; IV, indeterminate ventricle; LV, left ventricle; RV, right ventricle; V, ventricle. [partially modified from Ref. (12)]

*Univentricular* connection is present when the atria are connected predominantly with one ventricular chamber due to absence of one AV valve (absent connection) or to the presence of two valves or of a common valve predominately draining in one ventricle (double inlet connection) (Figure 2). In both conditions, one ventricle is of good size and the other is hypoplastic, missing the inlet portion, and the two ventricular cavities are separated by an interventricular septum.

The main ventricular chamber may be of right or left morphology or, in exceptional cases, indeterminate, which means that the ventricular septum failed to develop. Establishing the type of ventricular loop is a fundamental step since an absent AV connection may occur with discordant loop (l-loop in situs solitus, d-loop in situs inversus) so that the morphology of the main ventricular chamber may be right or left, respectively.

It should be underlined that, when an even tiny ventricular septum is recognizable, a second hypoplastic ventricular chamber is almost always existing and it is of right morphology, anteriorly located, when the main ventricular chamber is of left morphology, and it is posteriorly located and of left morphology when the mean ventricular chamber is of right morphology.

At the VA junction, the connection is concordant when the aorta takes origin from the left ventricle and the pulmonary artery from the right ventricle, or discordant when the aorta takes origin of the right ventricle and the pulmonary artery from the left ventricle. Double outlet VA connection is observed when more than one and half of the great arteries origin from a ventricle. The condition when only one patent great artery takes origin from the heart is called single outlet: single aortic outlet with pulmonary atresia, single pulmonary outlet with aortic atresia or single outlet with truncus arteriosus. Aortic atresia and pulmonary atresia with intact septum are usually characterized by the presence of one large main ventricle and of a hypoplastic accessory ventricle. In these cases, the AV connections are usually concordant and the ventricles have an inlet. However, aortic atresia can be associated with mitral atresia with severe hypoplastic left heart and absent inlet (absent left AV connection with single pulmonary outlet). Absent right AV connection with single aortic outlet, namely tricuspid–pulmonary atresia, is almost non-existing.

The hearts characterized by one large and one hypoplastic ventricular chamber are usually the consequence of univentricular AV connection (double inlet or absent AV connection) and are usually known as univentricular hearts.

But there are also hearts, with regular AV and VA connections presenting both inlet and outlet portions, in which one ventricle is unable to sustain the pulmonary or the systemic circulation. That is the case of hearts with hypoplastic left ventricle due to aortic stenosis/atresia or with hypoplastic right ventricle due to pulmonary stenosis/atresia or those with Ebstein anomaly and large atrialization of the right ventricular cavity.

Under the term “functionally univentricular heart,” Anderson and co-workers (14–18) unified different anatomical malformations characterized by the fact that one of the two ventricles is unable to sustain the pulmonary or the systemic circulation as a consequence of diminutive size or deficiency function. In these cases, the ventricles are not amenable for biventricular repair and only one ventricle or one and half ventricular repair is possible.

We reviewed our Anatomical Collection of CHD with the aim to identify the anatomical characteristics of the hearts corresponding to the new definition of functionally univentricular heart.

## Material and Methods

In the Anatomical Collection of the University of Padua, consisting in 1612 hearts with CHD, we identified the hearts corresponding to the definition of functionally univentricular heart.

Of each heart, we described the atrial situs, the AV, and VA connections, in terms of type and mode, the morphology of the ventricles and the associated anomalies.

On the base of the presence and morphology of the interventricular septum, each ventricle is reported as having right, left, or indeterminate morphology and on the base of dimensions as dominant or hypoplastic. The topology of the ventricles was identified as d- or l-loop: d-loop corresponding to morphologically right ventricle in right anterior position and morphologically left ventricle in left posterior position and l-loop with morphologically right ventricle in left anterior position and morphologically left ventricle in right posterior position. Mode of connection was used to identify the presence of two patent valves, common valve, imperforate valve, straddling, or overriding valves.

## Results

Two different groups of heart were identified with one large and one hypoplastic ventricles:

Hearts with univentricular AV connection:

- Double inlet left, right, or indeterminate ventricle,
- Absent left or right connection.

Hearts with biventricular AV connection and single VA connection:

- Aortic atresia with intact ventricular septum (IVS) and hypoplastic left ventricle;
- Pulmonary atresia with IVS and hypoplastic right ventricle.

## Hearts with Univentricular AV Connection

Univentricular AV connection was present in 196 (12%) cases. Fifty-five hearts showed a double inlet connection and 141 an absent AV connection (Table 1).

**Table 1:** **Univentricular AV connection: atrial situs**.

Atrial situs	Double inlet ventricles: 55 hearts			Absent AV connection: 141 hearts	
	Double inlet LV	Double inlet RV	Double inlet IV	Absent left connection	Absent right connection
Solitus	29	5	1	108	28
Right isomerism	6	12	–	–	–
Left isomerism	–	2	–	2	3
Total	**35**	**19**	**1**	**110**	**31**

*AV, atrioventricular; IV, indeterminate ventricle; LV, left ventricle; RV, right ventricle*.

The situs was solitus in 171 cases. Right isomerism was present in 18 and left isomerism in 7. No case of situs inversus was found (Table 1).

## Double Inlet Ventricle

Among the 55 hearts with double inlet connection, the left ventricle was the dominant ventricle in 35 cases and the right ventricle was the dominant ventricle in 19 cases (Table 1). In one case, only one ventricular chamber with indeterminate morphology was present (Table 1).

### Double inlet left ventricle

The atrial situs was solitus in 29 cases and a right atrial isomerism was present in 6. No case of double inlet left ventricle was found in association with situs inversus or left atrial isomerism (Table 1).

#### Hearts with situs solitus: 29 cases

The dominant ventricle showed a left ventricular morphology and the hypoplastic ventricle of right morphology was located antero superiorly and to the right (d-loop) in 23 and on the left (l-loop) in 6 hearts.

**Figure 3:**
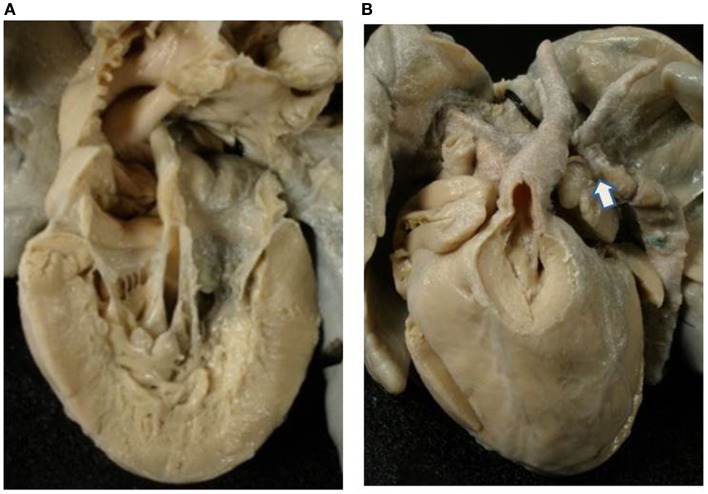
**Double inlet left ventricle**. **(A)** Both the AV valves drain into a posterior ventricular chamber of left morphology. **(B)** External view of the heart: the hypoplastic right ventricle is located in antero-superior position and gives origin to the aorta (discordant ventriculo-arterial connection). Note the restrictive ventricular septal defect (bulbo-ventricular foramen). The arrow indicates the surgical repair of the aortic arch obstruction.

Two separate valves (Figures 2C and 3) were present in 27 cases. An imperforate valve was noted in two cases: right in one and left in one. The right AV valve was straddling in 7 cases and overriding in one (Table 2).

**Table 2:** **Double inlet ventricle: mode of AV connection**.

Mode of AV connection	Double inlet LV 35 hearts		Double inlet RV 19 hearts			Double inlet IV 1 heart
	Situs	Right	Situs	Right	Left	Situs
	solitus	isomerism	solitus	isomerism	isomerism	solitus
	29	6	5	12	2	1
Two patent valves	27	–	2	1	–	1
Common valve	–	6	3	11	2	–
Imperforate right valve	1	–	–	–	–	–
Imperforate left valve	1	–	–	–	–	–

*AV, atrioventricular; IV, indeterminate ventricle; LV, left ventricle; RV, right ventricle*.

**Figure 4:**
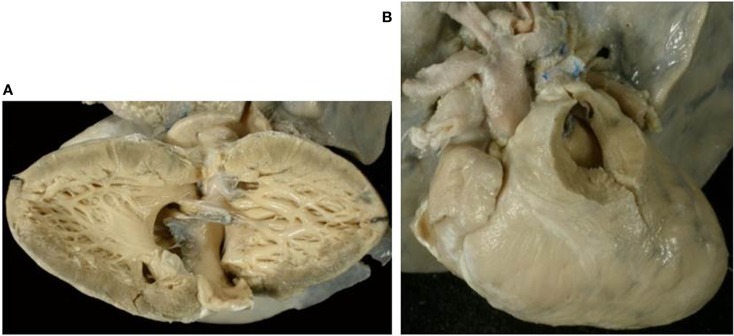
**Double inlet left ventricle with concordant ventriculo- arterial connection (Holmes heart)**. **(A)** View of the dominant left ventricle in which both the atria mostly drain through two patent AV valves. **(B)** External view of the heart with hypoplastic right ventricle and normally related great arteries. The aorta takes origin from the posterior ventricular chamber of left morphology and the pulmonary artery from the anterior hypoplastic ventricle of right morphology (concordant ventriculo-arterial connection).

The VA connections were concordant in 4 cases (Holmes heart) (Figure 4), discordant in 23 (Figure 3), and double outlet left ventricle in 1. Single aortic outlet (pulmonary atresia) was present in 1 case, with the aorta originating from the right ventricle (Table 3). In 3 cases an imperforate pulmonary valve was observed, 1 with concordant and 2 with discordant VA connections.

**Table 3:** **Double inlet ventricles: type of VA connection**.

Type of VA connection	Double inlet LV 36 hearts		Double inlet RV 19 hearts			Double inlet IV 1 heart
	Situs	Right	Situs	Right	Left	Situs
	solitus	isomerism	solitus	isomerism	isomerism	solitus
	29	6	5	12	2	1
Concordant	4	–	2	–	–	–
Discordant	23	1	–	–	–	–
Double outlet RV	–	–	1	7	1	–
Double outlet LV	1	1	–	–	–	–
Double outlet IV	–	–	–	–	–	1
Single Ao outlet from RV (Po atresia)	1	4	2	4	–	–
Single Po outlet from RV (Ao atresia)	–	–	–	1	1	–

*Ao, aortic; IV, indeterminate ventricle; LV, left ventricle; RV, right ventricle; Po, pulmonary; VA, ventriculo-arterial*.

The associated anomalies are described in Table 4.

**Table 4:** **Double inlet ventricle: associated anomalies**.

Associated anomalies	Double inlet LV 36 hearts		Double inlet RV 19 hearts			Double inlet IV 1 heart
	Situs	Right	Situs	Right	Left	Situs
	solitus	isomerism	solitus	isomerism	isomerism	solitus
	29	6	5	12	2	1
d-loop	23	4	4	11	2	–
l-loop	6	2	1	1	–	–
Dextrocardia	–	–	1	2	–	–
Appendages juxtaposition	1	–	–	–	1	–
Persistent LSVC to coronary sinus	1	–	1	–	2	–
Persistent LSVC to atrium	–	5	–	6	–	–
Interrupted IVC	–	–	–	–	2	–
Symmetric drainage of Po veins to atria	–	–	–	–	2	–
Total anomalous Po drainage into SVC	–	4	–	5	–	–
Total anomalous Po drainage into atria	–	2	–	4	–	–
Total anomalous Po drainage in IVC	–	2	–	–	–	–
Absent coronary sinus	–	6	1	12	–	–
ASD/PFO	14/15	2/−	2/1	7/−	–	−/1
Common atrium	–	4	2	5	2	–
Mitral valve cleft	3	1	–	1	–	–
Parachute mitral valve	1	–	–	–	–	–
Mitral double orifice	2	–	–	–	–	–
AV septal defect	–	6	3	11	2	–
Perimembranous VSD	8	–	2	–	–	–
Muscular VSD	21	1	–	1	–	–
Restrictive VSD	3	–	–	–	–	–
Sub aortic infundibulum	21	1	1	4	–	–
Bilateral infundibulum	3	4	3	7	1	1
Left outlet obstruction	5^a^	–	–	–	–	–
Right outlet obstruction	2^b^	–	1^a^	–	–	–
Bicuspid Po valve	2	–	1	–	1	–
Bicuspid aortic valve	3	1	–	–	–	–
Right aortic arch	–	3	–	6	1	1
Aortic arch coarctation	7	–	–	–	–	–
Aortic arch interruption	2	–	–	–	–	–
PDA (left/right)	17/−	3/3	3/−	5/1	1/−	–
Ductus dependent Po circulation	4	4	2	3	–	–
Systemic dependent Po circulation	–	–	–	1	–	–
Single coronary artery	–	1	1	–	–	–

*ASD, atrial septal defect; AV, atrioventricular; IV, indeterminate ventricle; IVC, inferior vena cava; LSVC, left superior vena cava; LV, left ventricle; PDA, patent ductus arteriosus; PFO, patent foramen ovalis; Po, pulmonary; RV, right ventricle*.

*^a^Fibrous diaphragm*.

*^b^Hypertrophy of septal bands*.

#### Hearts with right isomerism: six cases

Six cases presented with *right isomerism* in association with the absence of the spleen and visceral heterotaxy (Table 1).

A common AV valve was present in all cases (Table 2).

The VA connections were discordant in one case and double outlet from left ventricle in one. A single aortic outlet (pulmonary atresia) was present in four cases with aorta originating from the right ventricle (Table 3).

The ventricular topology was d-loop in four and l-loop in two hearts (Table 4).

Associated anomalies are described in Table 4.

### Double inlet right ventricle

Among the 19 hearts with double inlet right ventricle, the atrial situs was solitus in 5 cases, right isomerism in 12, and left isomerism in 2 (Table 1).

#### Hearts with situs solitus: five cases

**Figure 5:**
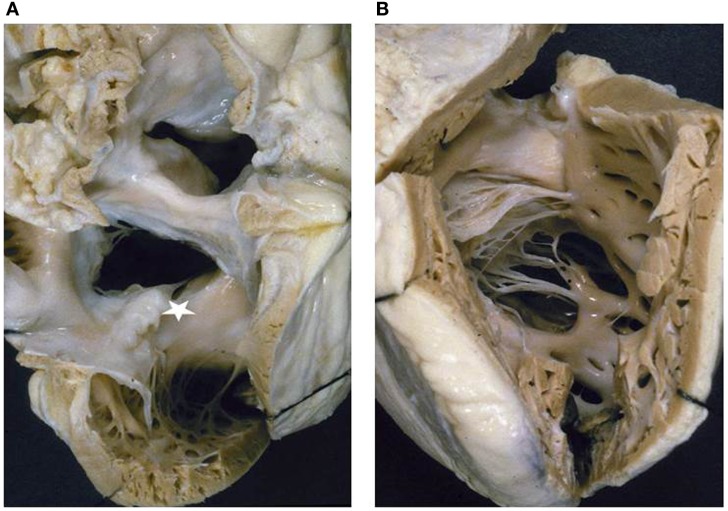
**Double inlet–outlet right ventricle**. **(A)** View from the atria: both the atria drain mostly into a main ventricular chamber of right morphology through two separate atrioventricular valves. The left atrioventricular valve straddles and overrides the interventricular septum (star): note the posterior small ventricular chamber of left morphology. **(B)** View of the large right ventricle with coarse trabeculations in which both the atrioventricular valves drain.

**Figure 6:**
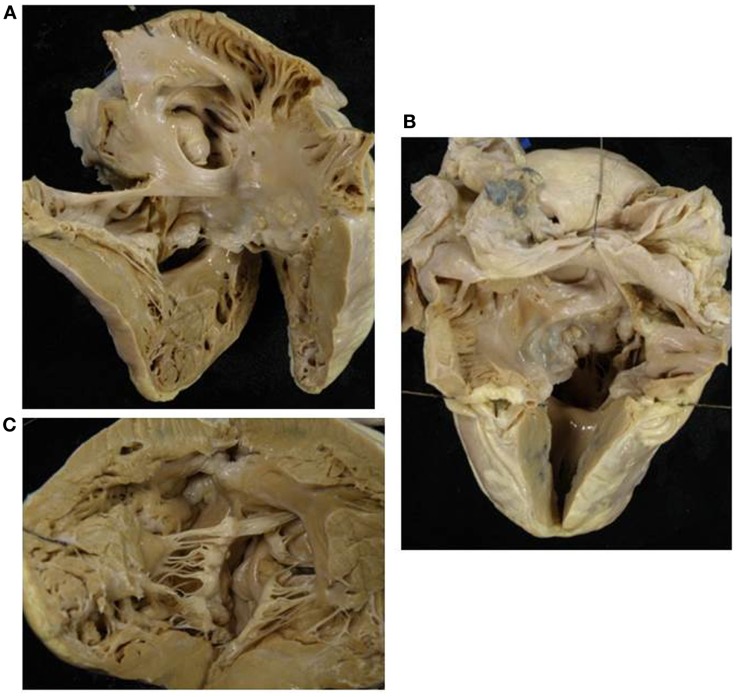
**Double inlet–outlet right ventricle**. **(A)** View of the right-sided cardiac chambers: a common atrioventricular valve is present and drains the blood from both atria, predominantly into an anterior ventricular chamber of right morphology. **(B)** View of the left-sided cardiac chambers: note the hypoplasia of the posterior left ventricle. **(C)** From the right ventricle both the aorta and the pulmonary artery take origin. The pulmonary outlet is stenotic. Note the common atrioventricular valve mostly connected to the right ventricle.

In these hearts, the dominant ventricle showed a right ventricular morphology with coarse apical trabeculations (Figures 5 and 6). The hypoplastic left ventricle was found in postero-inferior position on the left in four cases (d-loop) and on the right in one (l-loop) (Table 4).

Two separate valves were present in two cases (Figure 5) and a common AV valve in three (Figure 6) (Table 2).

The VA connections were concordant in two cases, double outlet right in one (Figure 6) and single aortic outlet (pulmonary atresia) from the right ventricle in two (Table 3).

The associated anomalies are reported in Table 4.

#### Hearts with right isomerism: 12 cases

In the 12 cases with right isomerism, a common AV valve was present in 11 cases and two separated valves in 1 (Table 2).

The VA connections were double outlet right ventricle in seven, single aortic outlet (pulmonary atresia) from the right ventricle in four, and single pulmonary outlet (aortic atresia) from the right ventricle in one (Table 3).

The left ventricle was hypoplastic in all cases and located in left posterior position in 11 cases (d-loop) and on the right posterior position (l-loop) in 1 case (Table 4).

In all cases the spleen was absent with visceral heterotaxy and presence of bilateral bronchi and lungs of right morphology.

The associated anomalies are described in Table 4.

#### Hearts with left isomerism: two cases

In both cases with left isomerism, a common AV valve was present (Table 2). The VA connections were double outlet right ventricle in one case and single pulmonary outlet (aortic atresia) from the right ventricle in one (Table 3).

The ventricular topology was d-loop in all cases (Table 4).

Common atrium, persistent left SVC, interruption of IVC with azygos continuation in right SVC and symmetric drainage of the pulmonary veins were present in both (Table 4).

### Double inlet indeterminate ventricle

**Figure 7:**
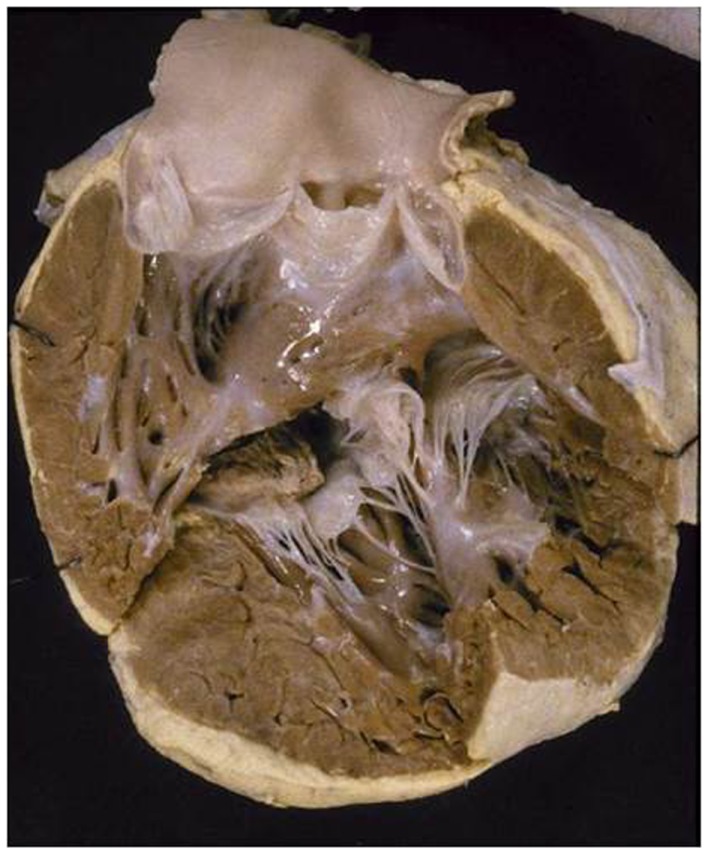
**Double inlet indeterminate ventricle**. Both the atrioventricular valves drain into a ventricular chamber with coarse trabeculations of indeterminate morphology. A second separate ventricle was not identified.

In this rare variant of double inlet ventricle, only a solitary chamber was found within the ventricular mass with coarse apical trabeculations (Figure 7). The existence of associated rudimentary ventricle was accurately excluded.

Two separate valves were present (Table 2) and the VA connections were double outlet from the indeterminate ventricular chamber (Table 3). A patent foramen ovalis was present at atrial level and bilateral infundibulum and a right aortic arch were also noted (Table 4).

## Hearts with Absent AV Connection

One hundred ten cases with absent left AV connection and 31 cases with absent right AV connection were found in our Anatomical Collection. The absent connection not always corresponded to mitral or tricuspid atresia because in some cases the development of l-ventricular loop placed the left ventricle on the right and the right ventricle on the left, so that the patent valve could be a mitral valve on the right and the tricuspid valve on the left.

### Absent left AV connection

One hundred eight hearts, characterized by the absence of the left AV valve, presented with situs solitus and only two with left isomerism (Table 1). In no case, absence of the left AV valve was found in association with situs inversus or right isomerism.

#### Hearts with situs solitus: 108 cases

**Figure 8:**
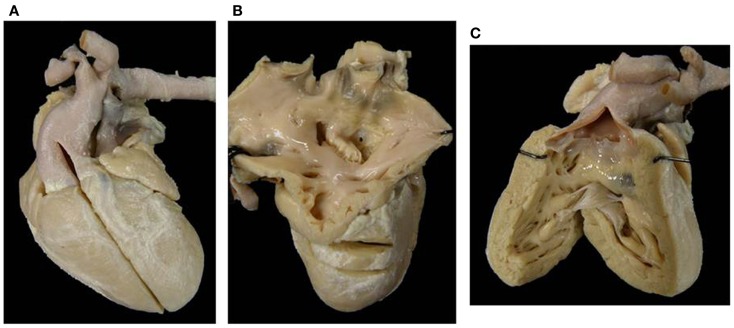
**Absent left atrioventricular connection (mitral atresia) with discordant ventriculo-arterial connection**. **(A)** External view of the heart with the aorta in right anterior position and the pulmonary artery in left posterior position. **(B)** At the floor of the left atrium, there is no orifice (absent left atrioventricular connection). **(C)** A good sized, right-sided, morphologically right ventricle (d-loop) is present from which the aorta takes origin (discordant ventriculo-arterial connection). The pulmonary artery takes origin from the left ventricle (not shown).

A regular d-ventricular loop was observed in 98 cases with left-sided hypoplastic left ventricle (Figures 2D and 8). In the 10 cases with l-ventricular loop and absent left AV connection the right-sided patent valve was the mitral and the hypoplastic ventricle was the right one.

##### Hearts with d-loop: 98 cases with dominant ventricle with right ventricular morphology (“mitral atresia”)

The left AV valve was absent in 92 cases and imperforated in 5. A common AV valve was present in 1 case (Table 5). Among the cases with absent left AV valve, the tricuspid valve was straddling in 2.

**Table 5:** **Absent AV connections: mode of AV connection**.

Mode of AV connection	Absent left AV connection 110 hearts				Absent right AV connection 31 hearts		
	Situs solitus 108 hearts		Left isomerism 2 hearts		Situs solitus 30 hearts		Left isomerism 1 heart
	d-loop 98	l-loop 10	d-loop 1	l-loop 1	d-loop 27	l-loop 3	l-loop 1
Absent left valve	92	10	1	1	–	–	–
Absent right valve	–	–	–	–	26	3	1
Common valve	1	–	–	–	–	–	–
Imperforate right valve	–	–	–	–	1	–	–
Imperforate left valve	5	–	–	–	–	–	–

*AV, atrioventricular*.

In 62 cases, a single pulmonary outlet (aortic atresia) with pulmonary artery originating from the right ventricle was present (Table 6). In the remaining, the VA connections were concordant in 11, discordant in 2 (Figure 8), double outlet from right ventricle in 19, and single aortic outlet (pulmonary atresia) with aorta originating from the right ventricle in 4 hearts (Table 6).

**Table 6:** **Absent AV connections: ventriculo-arterial connections**.

VA connections	Absent left AV connection 110 hearts				Absent right AV connection 31 hearts		
	Situs solitus		Left isomerism		Situs solitus		Left isomerism
	108 hearts		2 hearts		30 hearts		1 heart
	d-loop 98	l-loop 10	d-loop 1	l-loop 1	d-loop 27	l-loop 3	l-loop 1
Concordant	11	1	–	–	16	–	–
Discordant	2	6	–	1	5	1	–
Double outlet RV	19	–	–	–	–	1	1
Double outlet LV	–	2	–	–	–	–	–
Single Po outlet from RV (aortic atresia)	62	–	–	–	–	–	–
Single Ao outlet from RV (pulmonary atresia)	4	1	1	–	2	1	–
Single Ao outlet from LV (pulmonary atresia)	–	–	–	–	2	–	–
Single outlet truncus arteriosus	–	–	–	–	2	–	–

*Ao, aortic; AV, atrioventricular; LV, left ventricle; Po, pulmonary; RV, right ventricle; VA, ventriculo-arterial*.

The left ventricle was hypoplastic in all cases and slit-like in cases with associated aortic atresia. Fibroelastosis of the diminutive left ventricle was noted in two cases.

Detailed description of associated anomalies is reported in Table 7.

**Table 7:** **Absent AV connections: associated anomalies**.

Associated anomalies	Absent left AV connection 111 hearts				Absent right AV connection 31 hearts		
	Situs solitus		Left isomerism		Situs solitus		Left isomerism
	108 hearts		2 hearts		30 hearts		1 heart
	d-loop 98	l-loop 10	d-loop 1	l-loop 1	d-loop 27	l-loop 3	l-loop 1
Dextrocardia	–	–	1	1	6	1	1
Appendages juxtaposition	1	–	–	–	5	–	–
Persistent LSVC to coronary sinus	20	–	–	–	1	–	1
Persistent LSVC to the atrium	2	–	1	1	–	–	–
Interrupted IVC	–	–	–	1	–	–	1
Total anomalous Po drainage	5	–	–	–	–	–	–
ASD/PFO	25/61	7/3	–	−/1	20/7	2/1	1/−
Intact atrial septum	10	–	1	–	–	–	–
Common atrium	2	–	–	–	–	–	–
Absent coronary sinus	2	–	–	–	–	–	–
Mitral valve double orifice	–	–	–	–	1	–	
Perimembranous VSD	16	–	1	–	11	–	–
Muscular VSD	4	9	–	1	12	2	–
Multiple VSD	10	1	–	–	4	1	1
Sub Ao infundibulum	6	6	–	1	7	2	–
Bilateral infundibulum	8	3	–	–	2	1	–
Left outlet obstruction by fibrous AV tissue	2	2	–	–	–	–	–
Bicuspid Po valve	5	3	–	1	4	–	–
Bicuspid aortic valve	9	–	–	–	1	–	–
PDA (left/right)	93/1	7/−	–	–	17/−	2/−	0/1
Right aortic arch	1	–	1	1	1	–	1
Aortic arch coarctation	30	4	–	–	4	–	1
Retro esophageal subclavian artery	5	–	–	–	–	–	–
Single coronary artery	–	–	–	–	1	–	–
Ductus dependent Po circulation	–	1	–	–	–	–	–
Systemic dependent Po circulation	–	–	1	–	–	1	–

*ASD, atrial septal defect; AV, atrioventricular; IVC, inferior vena cava; LSVC, left superior vena cava; PFO, patent foramen ovalis; PDA, patent ductus arteriosus; Po, pulmonary; VSD, ventricular septal defect*.

##### Hearts with l-loop: 10 cases with dominant ventricle with left ventricular morphology (“tricuspid atresia”)

In all cases, the left AV valve was absent and the patent right AV valve was mitral in morphology. In one case, the right AV valve was straddling (Table 5).

The VA connections were concordant in one, discordant in six, double outlet from left ventricle in two, and single aortic outlet (pulmonary atresia) from right ventricle in one (Table 6).

Associated anomalies are found in Table 7.

#### Hearts with left isomerism: two cases

In both cases atria and lungs showed a bilateral left morphology (both left atrial appendages, lungs with two lobes and lingula), abdominal visceral heterotaxy, and multiple spleens.

A d-ventricular loop was noted in 1 case and l-loop in the other.

In the heart with dominant ventricle with right ventricular morphology (d-loop), the patent AV valve was the tricuspid valve (“mitral atresia”) and the VA connection was single aortic outlet (pulmonary atresia) from the right ventricle associated to dextrocardia (Tables 6 and 7).

In the heart, dominant ventricle with left ventricular morphology (l-loop) the patent AV valve was the mitral valve (“tricuspid atresia”). In this case, the VA connection was discordant and dextrocardia was present (Tables 6 and 7).

Associated anomalies in Table 7.

### Absent right AV connection

**Figure 9:**
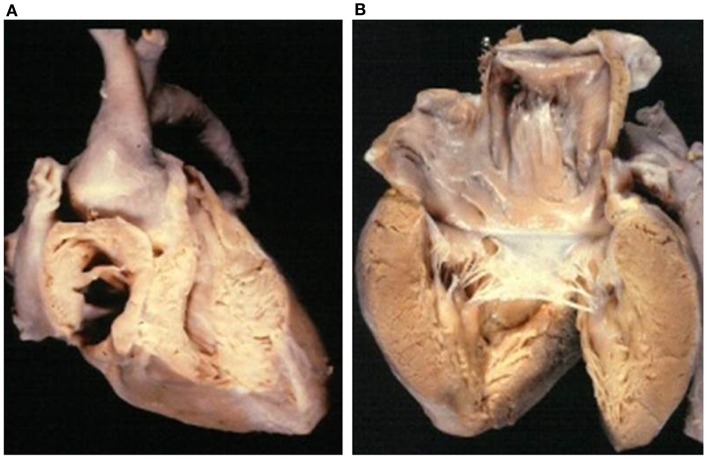
**Absent right AV connection (tricuspid atresia) with concordant VA connection**. **(A)** There is no connection between the right atrium and the underlying right ventricle. The diminutive right ventricle is located interiorly and on the right (d-loop) lacks of an inlet portion and gives origin to a hypoplastic pulmonary artery. The two ventricles communicate through a ventricular septal defect. **(B)** The left atrium drains through a mitral valve into the left ventricle.

**Figure 10:**
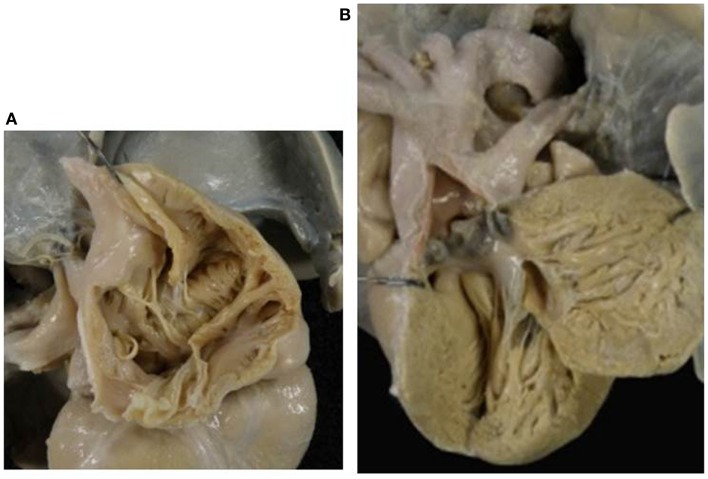
**Absent left AV valve (tricuspid atresia) with truncus arteriosus**. **(A)** There is no orifice in the floor of the right atrium. **(B)** A common arterial trunk arises from the heart, prevalently from the left ventricle, and gives origin to both systemic and pulmonary circulations. Note the dysplastic, tricuspid truncal valve.

**Figure 11:**
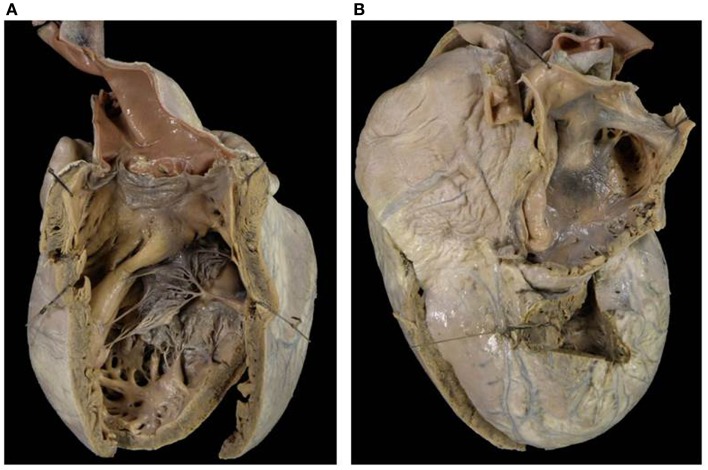
**Absent right atrioventricular connection in l-loop with single aortic outlet (pulmonary atresia)**. **(A)** A morphological tricuspid valve joins the left atrium with a main ventricular chamber of right morphology (l-loop), from which the aorta takes origin. The pulmonary valve is atretic (single aortic outlet). **(B)** At the floor of the right atrium there is no orifice. The underlying ventricular chamber is hypoplastic, posterior, right-sided and of left ventricular morphology (l-loop).

Thirty-one hearts with absent right AV connection were present in our Anatomical Collection (Figures 2A,B and 9–11). The atrial situs was solitus in 30 cases and a left isomerism was present in 1 case. No case of situs inversus or right isomerism was found (Table 1).

#### Heart with situs solitus: 30 cases

The relation of the ventricles was d-loop in 27 (Figure 9) and l-loop in 3 (Figure 11). In these latter cases, the patent left-sided AV valve was a tricuspid valve and the hypoplastic ventricle was the right-sided left ventricle.

##### Hearts with d-loop: 27 cases with dominant ventricle with left ventricular morphology (“tricuspid atresia”)

Absent right AV valve was noted in 26 cases (Figures 2B, 9, and 10) and an imperforate right AV valve in 1 (Table 5).

The VA connections were concordant in 16 cases (Figure 9) and discordant in 5. A single aortic outlet (pulmonary atresia) was noted in 4 cases with the aorta originating from the right ventricle in 2 and from the left ventricle in 2. In all 4 cases with pulmonary atresia, the lungs were supplied by the ductus arteriosus. In 2 cases, a common arterial trunk was present (Figure 10) (Table 6).

The associated anomalies are reported in Table 7.

##### Hearts with l-loop: three cases with dominant ventricle with right morphology (“mitral atresia”)

Absent right AV valve was noted in all cases (Figure 11) with straddling left AV valve in one case. The patent AV valve was tricuspid in morphology. The main chamber was of right morphology and the left ventricle was hypoplastic in all cases.

The VA connections were discordant in one, double outlet from right ventricle in one, and single aortic outlet (pulmonary atresia) from right ventricle in one (Figure 11) (Table 6).

Associated anomalies in Table 7.

#### Hearts with left isomerism: one case

In the single heart with *left isomerism*, a patent left-sided tricuspid valve was found at the AV junction (l-ventricular loop) and the VA connection was double outlet from right ventricle (Table 6).

Dextrocardia was present and the hypoplastic left ventricle was located anterior and on the right (l-ventricular loop). Heterotaxy of abdominal viscera and multiple spleens were present.

Other associated anomalies are described in Table 7.

## Hearts with Biventricular AV Connection and Single VA Connection

**Figure 12:**
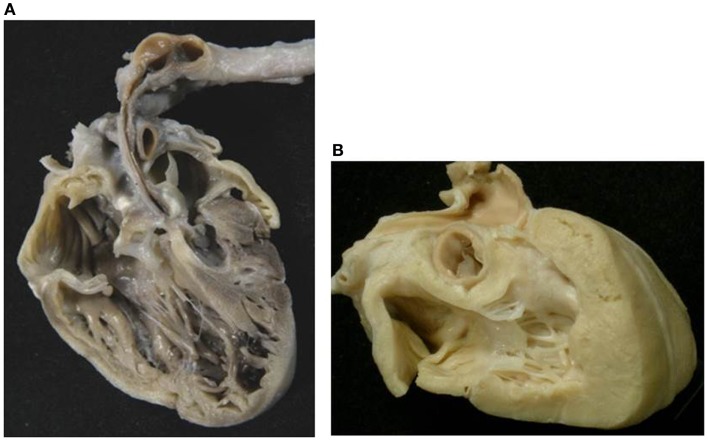
**Single outlet ventriculo-arterial connections with functionally univentricular heart**. **(A)** Aortic atresia with intact ventricular septum (single pulmonary outlet). Four chamber echocardiographic cut of the heart showing the severe hypoplasia of the left cardiac chambers. Note the tiny inlet portion of the left ventricle. This heart is only suitable for Norwood univentricular repair. **(B)** Pulmonary atresia with intact ventricular septum (single aortic outlet) with hypoplastic right ventricle. The size of the right ventricle is such that this heart may be suitable for one and half ventricle repair.

In our Anatomical Collection, 66 hearts were found with aortic atresia (single pulmonary outlet) with IVS (Figure 12A) and 58 with pulmonary atresia with IVS (single aortic outlet) (Figure 12B) (Table 8). Hearts with pulmonary atresia with VSD (tetralogy of Fallot with pulmonary atresia) (69 cases) and hearts with aortic atresia with VSD (9 cases) were excluded because they usually exhibit ventricles of good size.

**Table 8:** **Hearts with biventricular AV connection and single VA connection: mode of VA connection**.

Mode of VA	Aortic atresia + IVS	Pulmonary atresia + IVS	
connection	(single pulmonary outlet)	(single aortic outlet)	
	66 hearts	58 hearts	
		Hypoplastic	Dilated
		RV 51	RV 7
Absent pulmonary valve	–	25	4
Imperforate pulmonary valve	–	26	3
Absent aortic valve	61	–	–
Imperforate aortic valve	5	–	–

*AV, atrioventricular; IVS, intact ventricular septum; RV, right ventricle; VA, ventriculo-arterial*.

All cases with pulmonary or aortic atresia occurred in situs solitus and concordant AV connections.

## Aortic Atresia (Single Pulmonary Outlet)

In all the 66 hearts with aortic atresia with IVS, there was levocardia, d-loop topology of the ventricles with the hypoplastic left ventricle situated posterior and on the left (Figure 12A).

The aortic valve was absent in 61 cases and imperforate in 5 (Table 8).

The left ventricle was hypoplastic in all with fibroelastosis in 22 cases.

Associated anomalies are reported in Table 9.

**Table 9:** **Hearts with biventricular AV connection and single VA connection: associated anomalies**.

Associated anomalies	Aortic atresia + IVS (single pulmonary outlet) 66 hearts	Pulmonary atresia + IVS (single aortic outlet) 58 hearts	
		Hypoplastic	Dilated
		RV 51	RV 7
Persistent LSVC to coronary sinus	2	–	–
Anomalous Po drainage into RSVC	1	–	–
Intact atrial septum	2	–	–
ASD/PFO	24/37	22/29	7/−
Hypoplastic mitral valve	66	–	–
Mitral valve arcade	2	1	–
Dysplastic mitral valve	3	–	–
Hypoplastic tricuspid valve	–	30	–
Dysplastic tricuspid valve	–	16	–
Ebstein tricuspid valve	–	6	7
LV fibroelastosis	22	–	–
RV fibroelastosis	–	16	–
Bicuspid Po valve	1	–	–
Right aortic arch	–	1	–
Aortic arch coarctation	10	–	–
Retro esophageal subclavian artery	1	–	–
Single coronary artery	–	1	–
Ductus dependent Po circulation	–	51	7

*ASD, atrial septal defect; LV, left ventricle; AV, atrioventricular; PFO, patent foramen ovalis; IVS, intact ventricular septum; LSVC, left superior vena cava, RSVC, right superior vena cava VA, ventriculo-arterial; Po, pulmonary; RV, right ventricle*.

## Pulmonary Atresia (Single Aortic Outlet)

Fifty-eight hearts presented with pulmonary atresia with IVS. In all hearts, levocardia and d-loop were present.

**Figure 13:**
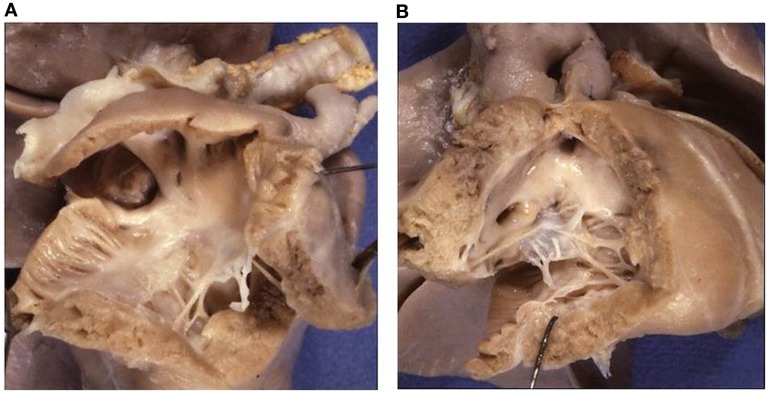
**Pulmonary atresia with intact ventricular septum (single aortic outlet)**. **(A)** View from the right cardiac chambers: the right ventricle is hypoplastic and hypertrophic. **(B)** View of the outflow of the same case: note the endocardial fibroelastosis of the diminutive right ventricle. This heart may be suitable for one and half ventricle repair.

Two different morphologic groups were found: one characterized by hypoplastic and hypertrophic right ventricle (Figures 12B, and 13) and one with dilated and functionally deficient right ventricle (Table 8).

### Heart with hypoplastic right ventricle: 51 cases

Among the 51 hearts with hypoplastic right ventricle (Tables 8 and 9), 14 cases presented all the ventricular components; in 12 the trabecular component was absent due to muscular hypertrophy; and in 23 cases both trabecular and outlet portions were not seen because of muscular hypertrophy.

The pulmonary orifice was absent (single aortic outlet) in 25 cases and an imperforated pulmonary valve (concordant VA connection) was present in 26 cases (Table 8).

The tricuspid valve was hypoplastic (Figures 12B and 13) in 30, dysplastic in 16 cases, and exhibited Ebstein malformation in 6.

Right ventricular fibroelastosis was noted in 16 hearts (Figure 13) and ventriculo-coronary artery connections in 10.

Other associated anomalies are reported in Table 9.

### Hearts with dilated right ventricle: Seven cases

All the seven hearts presented with extremely dilated right ventricle with thin parietal wall.

The pulmonary valve was absent in four and imperforate in three hearts (Table 8).

An Ebstein anomaly of the tricuspid valve was present in all cases.

Associated anomalies are reported in Table 9.

## Discussion

In the past, the normal ventricles were described to possess a sinus (inlet) and conus (outlet) (19, 20). Subsequently, they were considered to consist of three components: inlet, apical-trabecular, and outlet (21). The tripartite approach to the ventricular analysis and the morphological features of the apical-trabecular component of the interventricular septum nowadays permit to recognize right and left ventricular morphology also in hearts with abnormal AV connection.

The left ventricle is recognizable by its fine septal apical-trabecular trabeculations and the right ventricle by the coarse apical-trabecular aspect (11, 22). These anatomical characteristic are recognizable also in hearts that lack of inlet or outlet portions. The exception is the rare condition of a solitary or indeterminate ventricle in which there are no septal remnants and the apical component is uniformly coarse.

For long time, there was no agreement in considering as a true ventricle the ventricular chamber that lacks one component (23). Double inlet connection was named single ventricle despite, the fact that the heart possesses two ventricular chambers, one large and one small missing the inlet portion. The same consideration was applied to hearts showing tricuspid or mitral atresia (2, 23).

The hearts characterized by one large and one hypoplastic ventricular chamber are known with different terms such as: *cor triloculare biatriatum* (24–28), *single or common ventricle* (2, 19, 20, 29–34), *primitive ventricle* (35–37), *double inlet ventricle* (38–41), and *univentricular heart* (42–51).

All these definitions pointed the attention to the ventricular mass, identifying the morphology of the dominant ventricular chamber.

Afterward, the attention was focused not only to the ventricles but also to the AV connection and these hearts were regarded as having a *univentricular AV connection* (52–54). This term represents a common denomination for all the hearts in which both the atria, directly or indirectly, connect predominantly to only one ventricle. This fact does not exclude the presence of two ventricles, even if one is hypoplastic or incomplete. In this way, the concept of univentricular AV connection groups hearts with double inlet connection and hearts with absent right or left AV connection.

Recently, the term *functionally univentricular hearts* was introduced (14–18) to identify not only hearts with univentricular AV connection (absent right or left AV connection, double inlet left, right, or indeterminate ventricle), but also hearts with biventricular AV connection (each atrium connects separately with its own ventricle), in which the right or left ventricles are too small as to be unable to sustain the pulmonary or the systemic circulation, respectively, if a biventricular surgical repair is accomplished.

**Figure 14:**
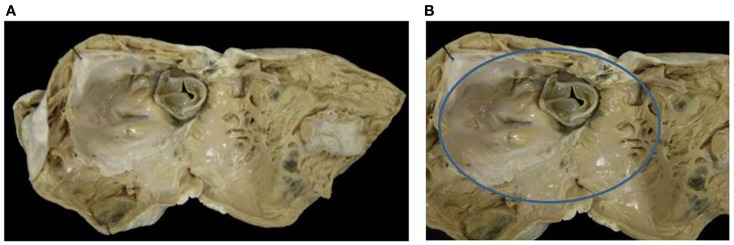
**Ebstein anomaly of the tricuspid valve**. **(A)** View from the apex of the right ventricle. A prosthetic valve was inserted ad the AV annulus. A large atrialized area is situated between the AV annulus and the displaced leaflets of the tricuspid valve. Note the thin wall of the right ventricle. In this patient, heart transplantation was performed because the dilated right ventricle was unable to sustain the pulmonary circulation. **(B)** Close up of the same specimen showing the atrialized area.

Even if a ventricle possesses all components, it does not mean that it will be always of size enough to bear a normal function. In pulmonary atresia with IVS, the right ventricle possesses all components but the ventricular cavity may be quite diminutive due to the extreme hypertrophy of the wall (55) (Figure 12B) or in some cases, with Ebstein anomaly of the tricuspid valve and large atrialization of a ventricular portion, the remaining right ventricle is functionally inadequate and unable to sustain a biventricular repair (Figure 14).

The surgical repair in functional univentricular hearts varies according to the morphology and the potential function of the hypoplastic ventricles. In some cases, with good sized ventricles, a biventricular correction can be performed, while in others the small ventricle can be suitable for a one and half ventricle repair. If one of the ventricle is severely hypoplastic or dysfunctional, only a univentricular repair can be considered.

*Double inlet left ventricle* with d-loop and concordant VA connection is a rare situation, known as Holmes heart (56, 57) (Figure 4). The most frequent VA connection is the discordant one (Figure 3). In these cases, the VSD (also known as bulbo-ventricular foramen) may be small and obstructive, accounting for subaortic stenosis in half of cases. Somebody avoid the use of the term bulbo-ventricular foramen in double inlet ventricle and suggest to describe the communication between the ventricular chambers simply as VSD (58, 59). The ascending aorta is hypoplastic compared with the dilated pulmonary artery and obstruction of the aortic arch, in the shape of coarctation, interruption or atresia, can be observed (Figure 3). In rare occasion, both great vessels may originate from the dominant left ventricle or from the hypoplastic right ventricle (double outlet left or right ventricle).

*Double inlet right ventricle* shows a dominant ventricle of right morphology with coarse apical trabeculations and a second hypoplastic ventricular chamber of left morphology located in postero-inferior position, usually on the left and rarely on the right, according to the ventricular loop.

In these cases right isomerism is frequently present and associated with a common AV valve as the mode of AV connection and double outlet right ventricle is the commonest VA connection (43, 49, 50). Any type of VA connection may occur: single aortic outlet (pulmonary atresia), concordant VA, and more rarely discordant VA connection.

In cases with right isomerism, the presence of common atrium, anomalous pulmonary venous drainage, and visceral heterotaxy can complicate the clinical presentation.

The description of the side of the absent connection (right or left), associated with the identification of the septal position of the ventricle (d- or l-loop) are to be preferred to the nomination of the malfomation as tricuspid in mitral atresia (60–62). In fact, in hearts with absent right AV connection and d-loop, the patent left AV valve is a mitral valve, but in case of l-loop, the patent left AV valve is a tricuspid valve (Figure 11).

*Absent right AV connection* (60) and d-loop is usually associated to concordant VA connection and the size of the right ventricle and pulmonary trunk depends on the dimension of the VSD. In some cases, the VA connection is discordant (transposition of the great arteries) and obstruction of the aortic arch can be present, if the VSD is restrictive. Dextrocardia and juxtaposition of the atrial appendages can be observed (63).

The majority of cases with *absent left AV connection* (60) is associated with single pulmonary outlet (aortic atresia). In these cases, the left ventricle is virtual. The rare presence of a VSD does not change the clinical presentation and double outlet right ventricle or discordant VA connections are found. Fibroelastosis of the left ventricle is not observed in absent left AV connection.

Hypoplastic left ventricle is associated to *isolated aortic atresia* with IVS. In these cases, the mitral valve is patent, but hypoplastic so that a tiny portion of the left ventricle is present, and fibroelastosis of the left heart is usually associated (Figure 12A).

**Figure 15:**
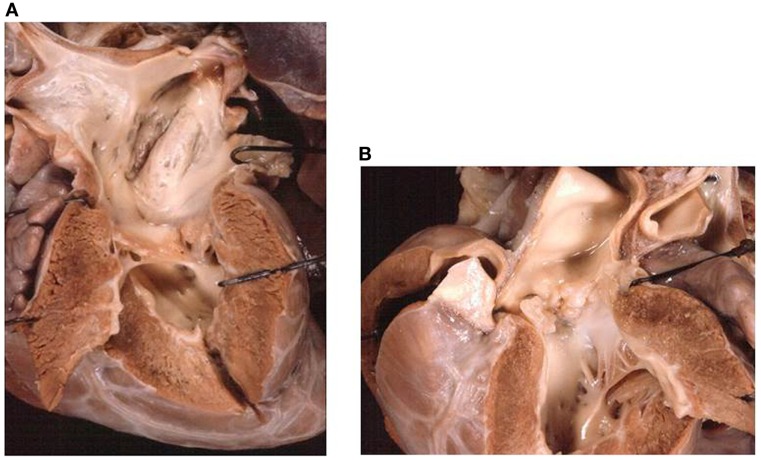
**Aortic stenosis with hypoplastic left heart**. **(A)** View from the left ventricular chambers: the left ventricle is hypoplastic with hypertrophic free wall. Note the hypoplastic mitral valve and the severe endocardial fibroelastosis of the left ventricle. **(B)** A severe aortic stenosis is present with dysplastic aortic valve. This heart may be amenable for one and half ventricle repair.

Also some cases with *critical aortic stenosis* may be associated with hypoplastic left ventricle and endocardial fibroelastosis (Figure 15). The patency of mitral or aortic valve does not change the physiology of the hypoplastic left heart, even though all its components are present. This is classical situation in which a one and half ventricle repair may be considered.

Also the right ventricle can be hypoplastic, not only in the setting of an absent right AV connection or double inlet left ventricle, but as consequence of pulmonary atresia or critical pulmonary stenosis with IVS. In cases with *pulmonary atresia* with IVS, the size of right ventricular cavity is quite reduced due to severe muscular hypertrophy of the free ventricular wall (Figure 12B) (55) and also the association with endocardial fibroelastosis (Figure 13) can jeopardize the ventricular contractility.

When pulmonary atresia is associated to VSD (Tetralogy of Fallot with pulmonary atresia) or aortic atresia is associated to VSD right and left ventricular cavities maintain a good size and biventricular repair can be performed.

In *Ebstein anomaly*, isolated or in association with pulmonary atresia with IVS (64), the extreme atrialization of the right ventricle is such that the residual ventricle is unable to sustain the pulmonary circulation if biventricular repair is accomplished, because the download displacement of septal and posterior leaflets of the tricuspid valve reduces the ventricle to apical and outlet portions (Figure 14).

In conclusion, the term functionally univentricular heart encompasses not only hearts known as single or common ventricle or univentricular heart, where one dominant and one hypoplastic chamber are present, and in which one ventricle lacks inlet component (double inlet or absent AV connection), but also hearts in which both ventricles possess all the morphological components but where one ventricle is tiny and unable to sustain the circulation.

The use of this definition, based not only on the morphology of the ventricles but also on their function, is relevant in planning biventricular, univentricular, or one and half ventricle surgical repairs (65–67).

## Conflict of Interest Statement

The authors declare that the research was conducted in the absence of any commercial or financial relationships that could be construed as a potential conflict of interest.
